# Fluid shear stress enhances natural killer cell's cytotoxicity toward circulating tumor cells through NKG2D-mediated mechanosensing

**DOI:** 10.1063/5.0156628

**Published:** 2023-08-11

**Authors:** Bing Hu, Ying Xin, Guanshuo Hu, Keming Li, Youhua Tan

**Affiliations:** 1 The Hong Kong Polytechnic University Shenzhen Research Institute, Shenzhen 518000, China; 2 Department of Biomedical Engineering, The Hong Kong Polytechnic University, Hong Kong 999077, China; 3 Research Institute for Smart Ageing, The Hong Kong Polytechnic University, Hong Kong 999077, China

## Abstract

Tumor cells metastasize to distant organs mainly via hematogenous dissemination, in which circulating tumor cells (CTCs) are relatively vulnerable, and eliminating these cells has great potential to prevent metastasis. In vasculature, natural killer (NK) cells are the major effector lymphocytes for efficient killing of CTCs under fluid shear stress (FSS), which is an important mechanical cue in tumor metastasis. However, the influence of FSS on the cytotoxicity of NK cells against CTCs remains elusive. We report that the death rate of CTCs under both NK cells and FSS is much higher than the combined death induced by either NK cells or FSS, suggesting that FSS may enhance NK cell's cytotoxicity. This death increment is elicited by shear-induced NK activation and granzyme B entry into target cells rather than the death ligand TRAIL or secreted cytokines TNF-α and IFN-γ. When NK cells form conjugates with CTCs or adhere to MICA-coated substrates, NK cell activating receptor NKG2D can directly sense FSS to induce NK activation and degranulation. These findings reveal the promotive effect of FSS on NK cell's cytotoxicity toward CTCs, thus providing new insight into immune surveillance of CTCs within circulation.

## INTRODUCTION

Tumor metastasis accounts for >90% of cancer-related deaths.^1,2^ The sequential steps of metastasis involve the detachment of a subpopulation of tumor cells from a primary tumor, invasion through the surrounding tissue, intravasation and survival in the vascular system, and extravasation into and generation of secondary tumors at distal sites. A failure at any step can undermine the successful establishment of metastatic tumors. Various rate limiting factors challenge the fate of disseminated tumor cells and the efficiency of the metastasis process (e.g., <0.01%).^3,4^ Upon entry into the vasculature, CTCs experience multiple environmental stresses, such as anoikis, FSS, oxidative stress, and immune surveillance.^1,5^ Only a small subpopulation of CTCs survive under these stresses to seed metastasis. The frequency of CTCs in vasculature is correlated with the prognosis and survival of cancer patients.^6,7^ Therefore, unveiling the combined effects of these critical factors on the survival of CTCs during hematogenous dissemination is important to facilitate the development of new therapeutic strategies for eradicating CTCs and preventing metastasis.

Physiological FSS varies according to locations (capillaries, veins, or arteries), ranging from 1 to 30 dyn/cm^2^.^8^ The influence of FSS on the survival of CTCs has been extensively investigated. For example, we have found that FSS eliminates the majority of suspended tumor cells in a magnitude- and time-dependent manner.^9,10^ Shear-induced cell death can be attributed to the elevated cell membrane damage,^11^ oxidative level,^12^ and sensitivity to cytokine-induced apoptosis.^13^ Nevertheless, certain tumor cells exhibit resistance to FSS-induced damage. For example, actomyosin is believed to be responsible for the survival of a subpopulation of CTCs in blood shear stress.^9,11^ Our previous research shows that CTCs can survive FSS-induced destruction through histone acetylation-mediated nuclear expansion.^14^ Furthermore, FSS can also promote the malignant functions of CTCs. For example, FSS facilitates epithelial–mesenchymal transition (EMT),^10,15^ stemness,^16^ and drug resistance of tumor cells in suspension.^17^

Immune system plays a major role in eliminating tumor cells at every step of tumor metastasis.^18^ Since the duration of CTCs in vasculature is relatively short (with a half-life of 1–2.4 h),^5,19–21^ effective killing of these tumor cells through immune responses requires direct and quick recognition and cytotoxicity. NK cells are an important part of the innate immune system and function as the major eliminator of CTCs because they can rapidly recognize and kill malignant cells through germ-line encoded activating receptors.^22,23^ The number and activation of NK cells are highly associated with the survival of cancer patients.^24–27^ The majority of NK cells in vasculature are CD56^dim^ subtype that has robust cytotoxicity with relatively low cytokine secretion ability compared with CD56^bright^ subtype.^28–30^ After conjugate formation through integrins like LFA-1,^31,32^ NK cells can be activated through the interaction with activating ligands, such as MICA/B and ULBP1–6, which directly induce target cell death by secreting perforin to form pores in target cell membrane and transport granzymes.^33–42^ In addition, NK cells can kill target cells through the death receptor ligand TRAIL^43,44^ and cytokines, such as TNF-α^45,46^ and IFN-γ.^47–49^

Recent evidence shows that mechanical cues, such as substrate stiffness and the membrane tension of target cells, have significant effect on NK cell functions. For example, fast actin dynamics regulate SHP-1 conformation and activity and promote NK cell activation.^50^ NK cells generate about 10 pN contractile force on MICA-coated nanowires, which is required for their activation.^51^ NK cells on MICA-coated substrates show stiffness-dependent activation and are most activated on 150 kPa surface that is much stiffer than normal human tissues.^52^ NK cells on NKp30 and anti-LFA-1 coated surface or beads secrete more cytokines and granzymes on stiff surfaces (142 kPa) or beads (264 kPa).^53^ NKG2D can discriminate different ligands through mechanically regulated ligand conformational changes using the biomembrane force probe and molecular dynamics,^54^ suggesting that NKG2D may be a potential mechanosensor.

In vasculature, CTCs are exposed to both NK cells and FSS simultaneously. However, their synergistic effects on the survival of CTCs remain unclear. In particular, whether FSS can influence NK cell's cytotoxicity against CTCs is still unknown. This study investigated the influence of FSS in vasculature on NK cell's cytotoxicity toward CTCs in a microfluidic system. The mechanisms underlying shear-dependent cytotoxicity were dissected, including NK secreted cytokines and granzyme B. Furthermore, the roles of NKG2D in shear-induced degranulation, NK activation, and the mediated cell death were examined. The mechanosensitivity of NKG2D in response to FSS was tested and the influence of FSS on NKG2D-mediated mechanosensing was explored.

## RESULTS

### FSS enhances NK cell's cytotoxicity toward CTCs

**FIG. 1.**
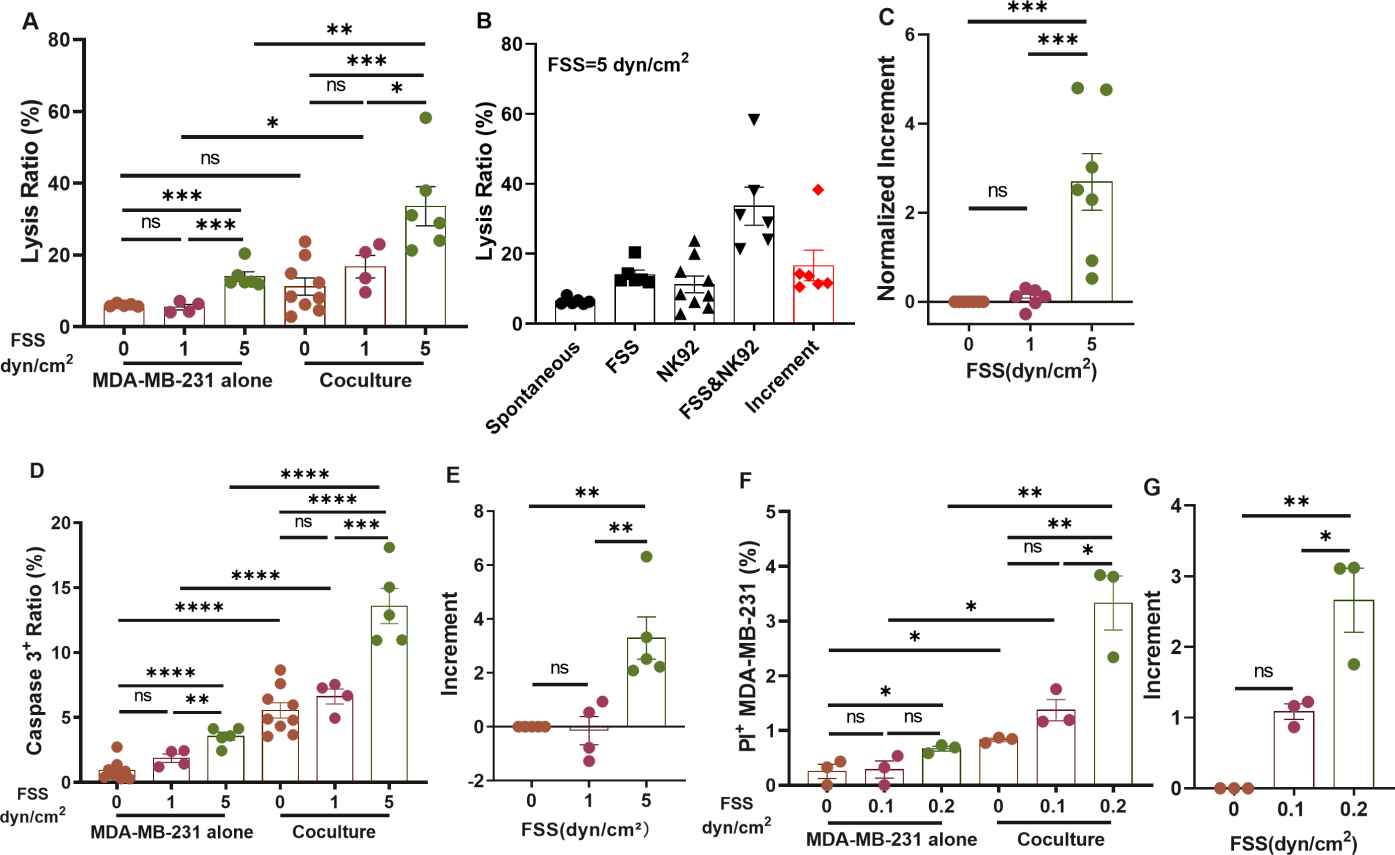
FSS enhances NK cell cytotoxicity toward breast CTCs. (a) Lysis ratio of MDA-MB-231 under 0, 1, and 5 dyn/cm^2^ FSS with or without NK92 co-culturing. n = 4 independent experiments. (b) Lysis ratio of MDA-MB-231 under no treatment (spontaneous), FSS (5 dyn/cm^2^), NK92, and FSS (5 dyn/cm^2^) and NK92. n = 6 independent experiments. (c) Normalized increment of tumor cell death at 0, 1, and 5 dyn/cm^2^ FSS. n = 4 independent experiments. Lysis ratio (LR) of tumor cells was calculated by LR=(1−survived target cell number/total target cell number)×100%; the increment was calculated by $\text{Increment}=({LR}_{{NK\_\mathrm{FSS}}^{}}-{LR}_{\mathrm{Spontaneous}})-({LR}_{{NK}^{}}-{LR}_{\mathrm{Spontaneous}})-({LR}_{{\mathrm{FSS}}^{}}-{LR}_{\mathrm{Spontaneous}})$; the normalized increment was calculated by Normalized Increment=Increment(LRNK−LRSpontaneous). (**d)** The percentage of caspase-3+ tumor cells under different magnitudes of FSS with or without NK92 co-culturing. n = 4 independent experiments. (e) Increment of caspase-3 activation at 0, 1, 5 dyn/cm^2^ FSS. n = 4 independent experiments. (f) Apoptotic ratio of MDA-MB-231 cells at 0, 0.1, and 0.2 dyn/cm^2^ FSS with or without NK92 co-culturing, indicated by the percentage of cells positive for propidium iodide (PI+). n = 3 independent experiments. (g) Increment of tumor cell death at 0, 0.1, and 0.2 dyn/cm^2^ FSS. n = 3 independent experiments. All the results were presented as mean ± SEM. The one-way ANOVA followed by Tukey's test was adopted for statistical analysis. NS, not significant; ^*^p < 0.05; ** p < 0.01; *** p < 0.001; and ^****^p < 0.0001.

To study the effect of FSS on NK cell's cytotoxicity, breast cancer cells in suspension were utilized to mimic CTCs and circulated with NK cells at the ratio of 1:4 under multiple levels of FSS (0, 1, 5, 10, and 20 dyn/cm^2^) within a microfluidic device.^9^ We found that 10 and 20 dyn/cm^2^ FSS induced substantial death of NK cells [Fig. S1(a)]. Thus, only 1 and 5 dyn/cm^2^ FSS were utilized in the rest of the study. Tumor cells exhibited a shear-dependent death response both with and without the co-culture with NK cells [Fig. 1(a)]. Importantly, the lysis ratio of tumor cells under both NK cells and FSS was much higher than the combined death induced by either NK cells or FSS, represented by the incremental lysis [Fig. 1(b), red bar]. In particular, under 5 dyn/cm^2^ FSS, the incremental lysis was more than twofold of that induced by NK cells [Fig. 1(c)], while this effect was negligible at 1 dyn/cm^2^. This indicates the reciprocal interaction between FSS and NK cells. Similarly, we also observed a shear-dependent caspase-3 activation and increment of caspase activity [Figs. 1(d) and 1(e)] in tumor cells, which might partially explain the shear-dependent lysis response [Figs. 1(a)–1(c)]. All the shear stresses above referred to the stress on the tubing wall. The shear stress distribution within the tubing depended on the radial position. Therefore, it was challenging to determine the exact FSS experienced by individual tumor cells. To validate this shear-induced response, suspended tumor cells were adhered to a poly-D-lysine (PDL)-coated chip and then treated with or without NK cells that were in suspension under 0, 0.1, and 0.2 dyn/cm^2^ FSS, respectively. In this scenario, all PDL-adhered tumor cells and the conjugated NK cells experienced a similar level of FSS on the chip, while suspended NK cells were circulated under the indicated wall shear stress that had minimal effect on their viability [Fig. S1(a)]. These PDL-adhered tumor cells showed as similar cytoskeleton, morphology, and focal adhesion as suspended cells under FSS [Figs. S1(b)–S1(g)]. The shear-dependent overall and incremental cell deaths of PDL-adhered tumor cells [Figs. 1(f), 1(g), and S1(h)] were similar to those of suspended tumor cells in the microfluidic tube [Figs. 1(a)–1(d)]. Taken together, these findings suggest that FSS within the vasculature can promote NK cell's cytotoxicity against CTCs.

### FSS promotes the entry of granzyme B into target cells

**FIG. 2.**
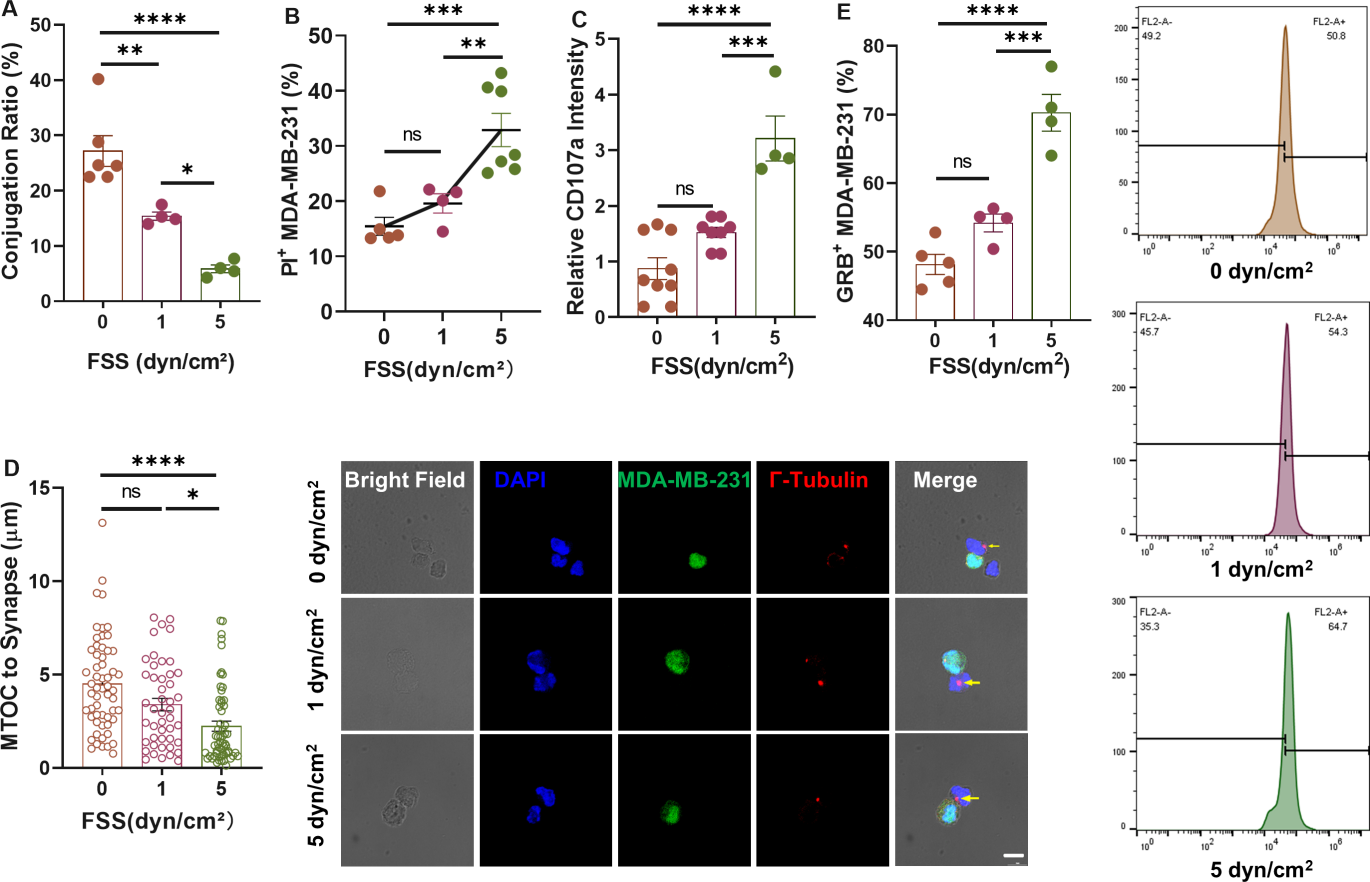
FSS promotes the entry of granzyme B into target cells. (a) Conjugate formation ratio between NK92 cells and MDA-MB-231 cells under different levels of FSS. n = 4 independent experiments. (b) The percentage of PI+ MDA-MB-231 cells within tumor cell-NK conjugates. n = 4 independent experiments. (c) The relative intensity of CD107a in NK cells co-cultured with tumor cells under different levels of FSS. n = 3 independent experiments. (d) MTOC polarization under different FSS represented by the distance between MTOC and the immunological synapse. n = 57, 46, and 59 cells for 0, 1, and 5 dyn/cm^2^ FSS. Scale bar: 10 *μ*m. (e) The percentage of Granzyme B+ MDA-MB-231 after co-culturing with NK92 under different FSS. The representative flow cytometry images were shown on the right panel. n = 4 independent experiments. Statistical analysis was performed using one-way ANOVA followed by Tukey's test (a)–(c) and (e) and Kruskal–Wallis test followed by Dunn's test (d). NS, not significant; ^*^p < 0.05; ^**^p < 0.01; ^***^p < 0.001; and ^****^p < 0.0001.

The major way by which NK cells kill CTCs is by forming conjugates with target cells, secreting perforin through the immune synapse to form pores on the cell membrane, and delivering the major effector enzyme granzyme B.^39–42,55^ To elucidate how FSS enhanced NK cell's cytotoxicity, we first tested the influence of FSS on conjugate formation. The results showed that the conjugate formation between tumor cells and NK cells decreased along with the increase in FSS [Fig. 2(a)], which might result from shear-mediated disturbance. Nevertheless, there were more apoptotic cells in the formed conjugates under higher FSS [Fig. 2(b)]. Furthermore, the expression of CD107a, a known marker of NK cell activity, significantly increased at 5 dyn/cm^2^ FSS [Fig. 2(c)], suggesting the shear-induced activation of NK cells. Microtubule organizing center (MTOC) of NK cells is responsible for the directed delivery of lytic granules.^41,56,57^ The distance between MTOC and the synapse decreased along with the increase in FSS [Fig. 2(d)], indicating the shear-dependent MTOC polarization. Under 5 dyn/cm^2^ FSS, the MTOCs were much closer to the synapses [Fig. 2(d)], suggesting the activation of the conjugated NK cells. As a consequence, more granzyme B was delivered into the conjugated MDA-MB-231 cells under 5 dyn/cm^2^ FSS [Figs. 2(e) and S2(g)], which might explain the enhanced NK cell's cytotoxicity toward CTCs.

Alternatively, NK cells can also induce tumor cell death via secreting cytokines, including TNF-α^45,46^ and IFN-γ,^47–49^ and via tumor necrosis factor-related apoptosis-inducing ligand (TRAIL).^13,41,58,59^ To investigate the roles of these mechanisms, the influence of FSS and tumor cells on the secretion of TNF-α and IFN-γ in NK cells was examined. There was no significant difference in TNF-α secretion when different levels of FSS were exerted with and without tumor cells [Fig. S2(a)]. Interestingly, the secreted IFN-γ decreased at 1 and 5 dyn/cm^2^ FSS compared to 0 dyn/cm^2^ [Fig. S2(c)]. These findings suggest that FSS does not promote the secretion of TNF-α and IFN-γ. This might be because cytokine secretion is different from cytotoxic granules secretion in NK cells.^60^ We further tested the cytotoxic effect of these cytokines under FSS. To treat MDA-MB-231 cells with or without FSS, 30 pg/mL TNF-α or 70 pg/mL IFN-γ was used. The results showed that there was no difference in cell apoptosis when suspended tumor cells were circulated under FSS with or without TNF-α and IFN-γ [Figs. S2(b) and S2(D)]. Furthermore, the blocking antibody was adopted to inhibit the functions of TRAIL. However, no significant difference was observed in tumor cell death under FSS compared with control IgG [Fig. S2 (e)]. In addition, the conditioned medium was collected from the co-culture of suspended tumor cells and NK cells under FSS. There was no net increment of cell lysis under 1 and 5 dyn/cm^2^ FSS [Fig. S2(f)], indicating that the cytokines secreted by NK cells may not contribute to the shear-induced increase in NK cell cytotoxicity.

Taken together, FSS impairs conjugate formation between NK and tumor cells while promoting NK cell activation and the delivery of granzyme B into target cells within the conjugates, which may enhance NK cell's cytotoxicity.

### FSS promotes granzyme B entry into target cells via NKG2D

**FIG. 3.**
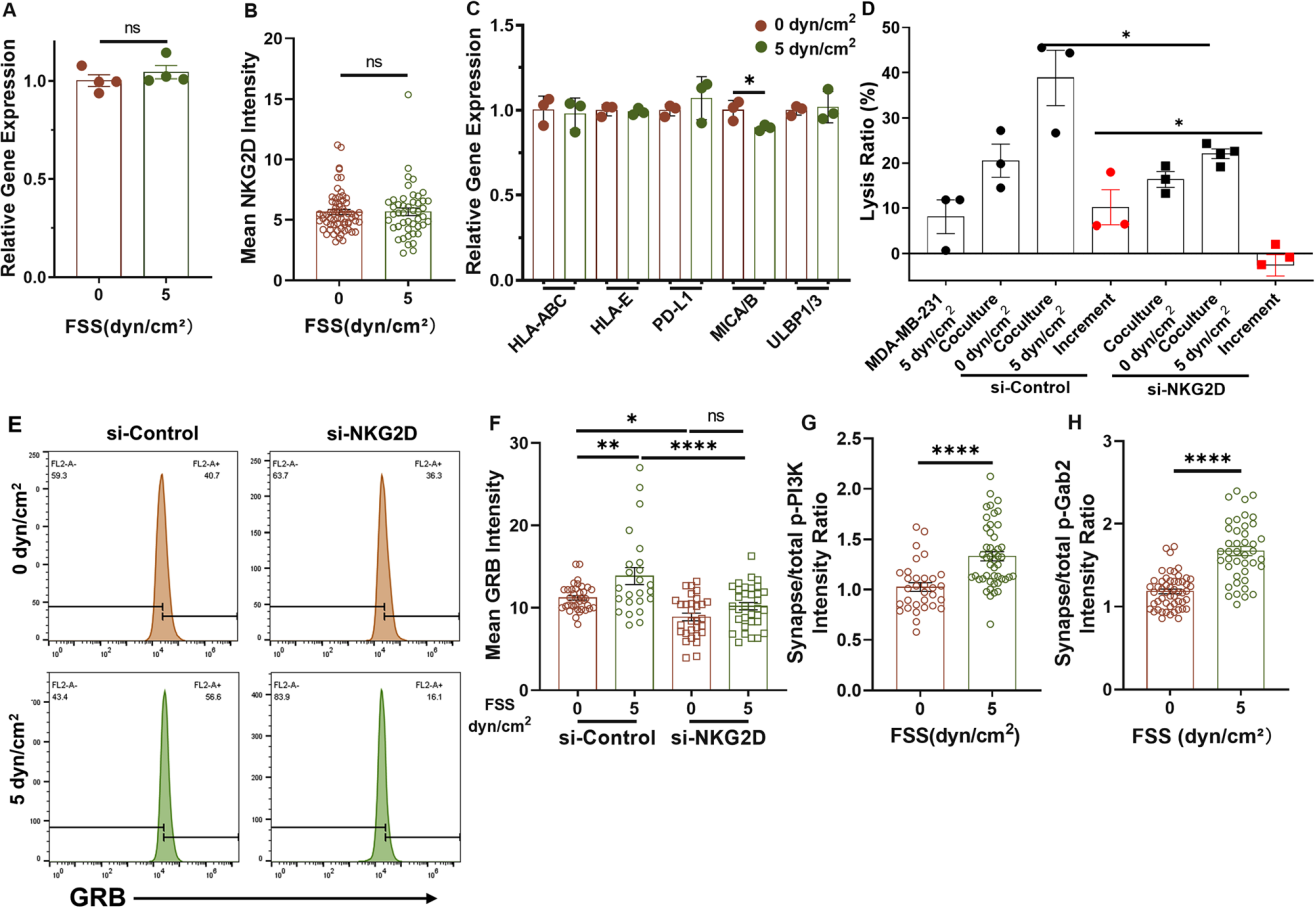
FSS promotes Granzyme B entry into target cells via NKG2D. (a) and (b) The expression of NKG2D at mRNA and protein levels in NK cells after shear treatment. NK cells were treated under 0 or 5 dyn/cm^2^ FSS for 4 h, and then analyzed for NKG2D expression at both mRNA (a) and protein (b) levels by qPCR and immunofluorescence staining, respectively. n = 4 independent experiments in (a); n > 46 cells in (b). (c) mRNA expressions of activating/inhibitory receptors in MDA-MB-231 under 0 or 5 dyn/cm^2^ FSS treatment for 4 h. n = 3 independent experiments. (d) Lysis ratio of MDA-MB-231 under both NK cells and 0 or 5 dyn/cm^2^ FSS after knocking down NKG2D. n = 3 independent experiments. (e) and (f) Granzyme B entry into MDA-MB-231 after co-culturing with NK92 when NKG2D was knocked down under 0 or 5 dyn/cm^2^ FSS. MDA-MB-231 was co-cultured with NK92 that were transfected with NKG2D siRNA or negative control and then treated under 0 or 5 dyn/cm^2^ FSS. The amount of Granzyme B within tumor cells was measured by flow cytometry (e) and immunofluorescence staining (f) in the conjugated cells. Representative of two independent experiments in (e). n > 30 cells in (f). (g) and (h) The relative level of phosphorylated PI3K and Gab2 between the synapse and the whole NK cell after co-culturing with MDA-MB-231 under 0 and 5 dyn/cm^2^ FSS. n > 32 cells in (g); n > 42 cells in (h). Statistical analysis was performed using unpaired t-test (a), (c), and (h), Mann–Whitney U-test (b) and (g), and one-way ANOVA followed by Tukey's test (d) and (f). NS, not significant; ^*^p < 0.05; ^**^p < 0.01; and ^****^p < 0.0001.

After engaging with target cells, the activity of NK cells is determined by a balance of activating and inhibitory signals.^61–63^ NK cells express germline-encoded receptors that recognize the corresponding ligands on the membranes of transformed cells.^62^ NKG2D, an important activating receptor expressed on NK cells, binds to several ligands that are structurally related to MHC class I, such as MICA, MICB, and ULBPs in humans.^64–67^ To test the role of NKG2D in shear-induced NK cell's cytotoxicity, we first explored the influence of FSS on the expressions of NKG2D and its ligands. Interestingly, shear treatment did not have significant impact on the mRNA and protein levels of NKG2D in NK cells [Figs. 3(a) and 3(b)] and ligands on MDA-MB-231 cells [Fig. 3(c)]. We next knocked down NKG2D in NK cells by electroporation [Fig. S3(a)] and found that silencing NKG2D significantly decreased the cytotoxicity increment under 5 dyn/cm^2^ FSS [Fig. 3(d)]. The amount of granzyme B in all tumor cells and the conjugated ones was remarkably elevated under FSS, which was blunted by the inhibition of NKG2D [Figs. 3(e) and 3(f)]. After ligation with MICA on target cells, PI3K and VAV1-Grb2 will be recruited to the intracellular domain of NKG2D with the help of the adaptor protein DAP10^68–71^ and Gab2^72^ to transduce activating signals, which then facilitate the delivery of granzyme B into target cells. We then examined the activation of PI3K and Gab2 signaling under FSS. The shear treatment promoted the phosphorylation levels of both PI3K and Gab2 within the synapse region under 5 dyn/cm^2^ FSS compared with the static condition [Figs. 3(g), 3(h) and S3(b) and S3(g)]. Pharmacologic inhibition of PI3K and VAV1 (downstream of Gab2) diminished the shear-induced increase in granzyme B delivery into tumor cells [Figs. S3(c)–S3(f)]. Together, these results suggest that FSS may promote NK cell's cytotoxicity through NKG2D-mediated granzyme B injection.

### NKG2D is mechanosensitive to FSS

**FIG. 4.**
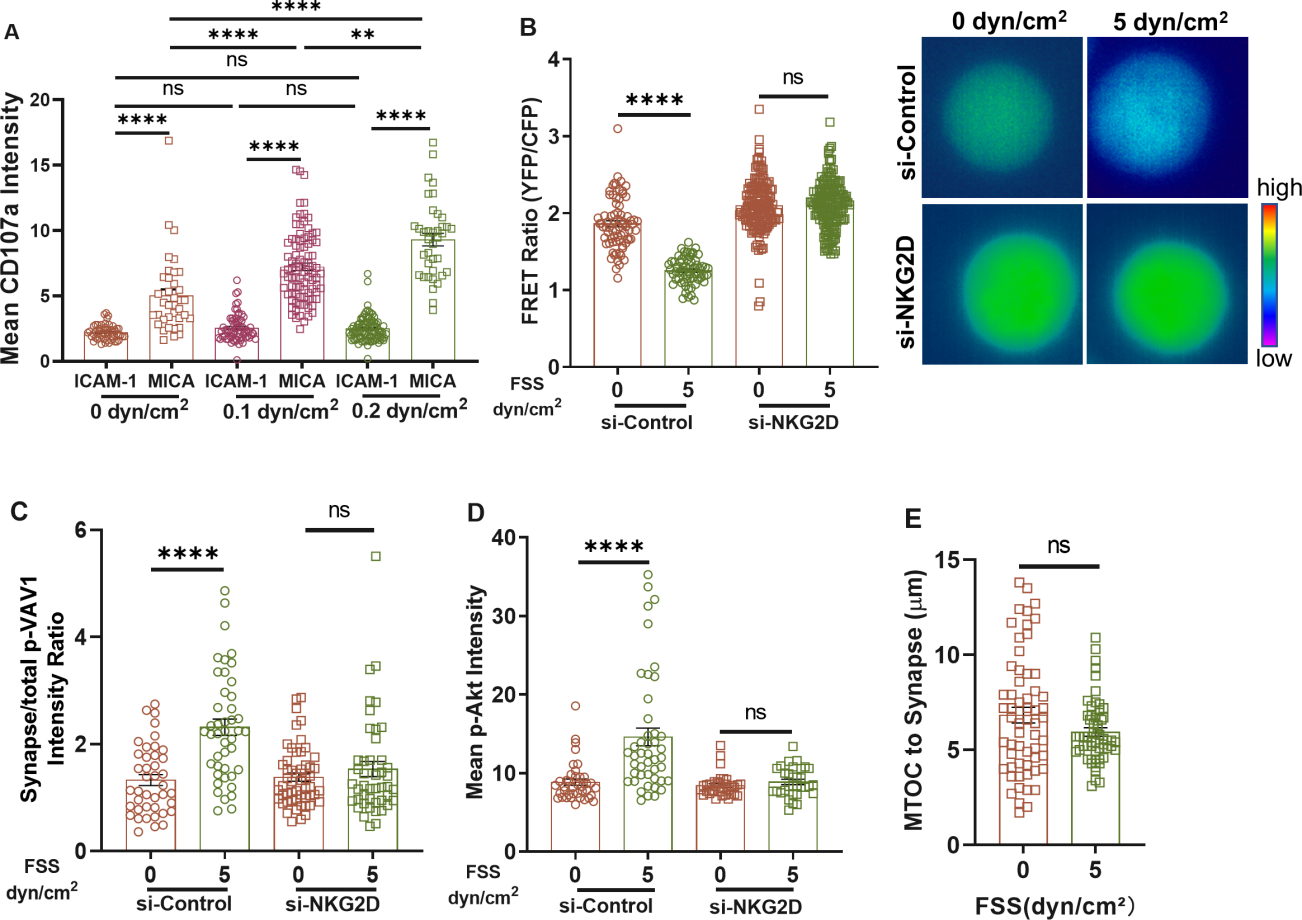
NKG2D is mechanosensitive to FSS. (a) CD107a expression in NK92 cells under different FSS when adhering to chips coated with MICA or ICAM-1. n > 40 cells. (b) FRET ratio (YFP/CFP) of NK92 cells when co-cultured with MDA-MB-231 cells under different FSS. Higher FRET ratio represents lower Lck activity. n > 64 cells. Right: representative FRET images. (c) The effect of NKG2D on phosphorylated VAV1 within the synapse of NK92 cells when co-cultured with MDA-MB-231 cells under different FSS. n > 41 cells. (d) The effect of NKG2D on phosphorylated Akt level of NK92 cells when co-cultured with MDA-MB-231 cells under different FSS. n > 32 cells. (e) MOTC polarization in NK92 cells when co-cultured with MDA-MB-231 cells under different FSS after knocking down NKG2D. n > 56 cells. Statistical analysis was performed by using Kruskal–Wallis test followed by Dunn's test (a), (c), and (d), one-way ANOVA followed by Tukey's test (b), and Mann–Whitney U-test (e). NS, not significant; ^*^p < 0.05; ^**^p < 0.01; ^***^p < 0.001; and ^****^p < 0.0001.

Our findings have implicated the involvement of NKG2D in the shear-induced activation of NK cells. We further explored whether NKG2D could directly sense and respond to FSS. Toward this goal, the surface of the microfluidic chip was coated with MICA, a known ligand to interact with NKG2D. NK cells were allowed to adhere to the surface and then treated under FSS. Along with the increase in FSS, adhered NK cells showed elevated expression of CD107a on the cell membrane [Fig. 4(a)], which indicated the enhanced cytotoxicity of NK cells. However, FSS could not promote CD107a expression in NK cells that adhered to the substrates coated with ICAM-1, ligand of integrin LFA-1. These findings indicate that the shear-induced activation of NK cells may be mediated through direct NKG2D-MICA force sensing. Upon the interaction with MICA, DAP10, the adaptor of NKG2D intracellular domain, recruits Grb2-VAV1 and PI3K to promote immune synapse formation and NK cell activation.^71,73^ Lck has been proposed to phosphorylate both VAV1^74,75^ and PI3K^76,77^ to initiate the downstream signaling. By using a fluorescence resonance energy transfer (FRET) sensor that could measure Lck activity, we found that FSS could promote Lck activity after NK cells were conjugated with target cells. Note that high FRET ratio of the sensor represented low Lck activity. Silencing NKG2D diminished the decrease in the FRET ratio and rescued Lck activity to the control level [Fig. 4(b)]. FSS also promoted the accumulation of phosphorylated VAV1 within the immune synapse, which was fully blunted by silencing NKG2D [Fig. 4(c)]. PI3K is another kinase phosphorylated by Lck. We found that the phosphorylation of Akt, downstream of PI3K, was significantly enhanced under FSS, and this increase was rescued by knocking down NKG2D [Figs. 4(d) and S3(h)]. Furthermore, FSS could promote the MTOC polarization of NK cells after conjugating with target cells [Fig. 2(d)]. Silencing NKG2D diminished this shear-induced MTOC polarization [Figs. 4(e) and S3(i)]. Taken together, these results demonstrate that the activating receptor NKG2D can directly sense and respond to FSS by activating the downstream Lck/Grb2/VAV1/PI3K signaling after the engagement with MICA, which may promote NK cell's cytotoxicity.

## DISCUSSION

Tumor metastasis is a complex and inefficient process. Within the circulation system, a small subset of CTCs can survive the challenging environment, such as anchorage-dependent anoikis, oxidative stress, immune surveillance, and FSS. Less than 0.01% of these cells may eventually seed metastatic tumors.^4,78^ Previous studies have found that FSS can effectively eliminate CTCs in a time-dependent manner,^9,10,12,79,80^ which may be compromised by the short half-life of CTCs in vasculature (∼1–2.4 h).^5,19–21^ Furthermore, the immune system is predetermined to kill tumor cells and the eradication of CTCs within vasculature requires rapid activation of the immune responses. As an essential part of the innate immune system, NK cells function as a major effector of CTC elimination. The primary tumor microenvironment has been reported to inhibit the infiltration and cytotoxicity of NK cells.^81–83^ Previous studies have shown that stiff substrates promote MTOC polarization and NK cell degranulation,^53^ indicating the potential of mechanical stimulations in regulating NK cell function. FSS is one mechanical cue that exists at varying levels in the vasculature but at very low level in primary tumor sites. However, whether FSS can influence NK cell function is still unknown.

We report here for the first time that FSS of physiologic magnitude can promote NK cell's cytotoxicity to CTCs within a relatively short duration. It is known that NK cells can induce tumor cell death through both direct and indirect interactions. In this study, we show that FSS has no significant impact on the secretion of TNF-α and IFN-γ in NK cells and does not enhance the sensitivity of tumor cells to the death induced by these cytokines, TRAIL, and the conditioned medium. This finding is different from the previous study that the death receptor ligand TRAIL can sensitize CTCs to FSS-induced cell death,^13^ which may be due to the fact that the amount of TRAIL secreted by NK cells is insufficient in mediating tumor cell death. Instead, FSS promotes NK cell degranulation and the delivery of granzyme B into target cells, suggesting the enhanced cytotoxicity. Thus, it is possible that one shear-activated NK cell may kill the conjugated tumor cell within a shorter time duration than a control NK cell. After efficient eradication, it may dissociate from the dead cell and continue to form conjugates with another tumor cell. Therefore, one NK cell may kill more tumor cells than one control cell within a given time duration under FSS, although shear treatment decreases the conjugate formation. Importantly, shear-induced NK activation is mediated by the mechanosensing of NKG2D-MICA interaction, inhibition of which abolishes the incremental effect of FSS on NK cell's cytotoxicity. Our findings provide new evidence to support that NK cells are sensitive to shear stress via NKG2D-mediated mechanotransduction, which may depend on force-induced alterations in ligand conformation.^54^

Previous research shows that cytotoxic T cells preferentially kill stiff tumor cells while sparing soft ones through perforin-mediated membrane pore formation.^84^ Malignant tumor cells are soft and enriched in cholesterol, which can protect them from cytotoxic T cell-mediated immunotherapy.^85^ Our previous studies find that CTCs that can survive after shear treatment exhibit much lower stiffness than control tumor cells,^86^ and the tumor cells selected by 3D soft fibrin are soft and malignant. These soft tumor cells may be thus more resistant to NK or T cell-mediated immune surveillance. Although FSS promotes NK cell's cytotoxicity, it is possible that a small subpopulation of CTCs with low stiffness but high resistance to both FSS and NK cells may still persist within the vasculature and eventually generate secondary lesions. Therefore, further investigation is needed to decipher the detailed survival mechanisms, which will facilitate the development of effective therapeutic strategies that can efficiently eliminate these resistant but malignant CTCs.

## CONCLUSIONS

In summary, this study reports that FSS enhances NK cell's cytotoxicity to CTCs in a shear-dependent manner. The incremental tumor cell death is attributed to the shear-induced activation of NK cells, which then deliver more granzyme B into CTCs to induce apoptosis, while the roles of the secreted cytokines and death ligands are dispensable. Importantly, when binding to the ligand MICA, NKG2D can directly sense FSS to promote NK cell activation and cytotoxicity. Silencing NKG2D abolishes FSS-mediated NK cell activation, granzyme B entry, and the incremental death of CTCs. These findings unveil a mechanically regulated CTC killing mechanism of NK cells, which may provide a novel mechanotargeting strategy for NK cell-based immunotherapy.

## METHODS

### Cell culture

NK92 cell line was a kind gift from Prof. Lei Sun in Hong Kong Polytechnic University (Table I). MDA-MB-231 cell line was purchased from ATCC. NK92 cells were cultured in Minimum Essential Medium α (Gibco) supplemented with 12.5% fetal bovine serum (Gibco), 12.5% horse bovine serum (Sigma), 0.2 mM inositol (Sigma), 0.1 mM 2-mercaptoethanol (Sigma), 0.02 mM folic acid (Sigma), 100 U/ml recombinant IL-2 (PeproTech), and 1% penicillin/streptomycin (Gibco) in an atmosphere of 5% CO_2_ at 37 °C. MDA-MB-231 cells were cultured in Dulbecco's Modified Eagle Medium (Gibco) supplemented with 10% fetal bovine serum and 1% penicillin/streptomycin in an atmosphere of 5% CO_2_ at 37 °C. Cells were passaged every 2–3 days using 0.25% Trypsin (Gibco). In some experiments, NK92 cells were pretreated with 50 μM PI3K inhibitor LY294002 for 30 min or with 5 *μ*M VAV1 inhibitor Azathioprine for 3 days.

**TABLE I.** Antibodies, recombinant DNAs, and reagents.

Reagent or resource	Source	Identifier
Antibodies		
Mouse monoclonal anti-human granzyme B-PE antibody	eBioscience	Cat#12-8896-42
Mouse monoclonal anti-human CD107a-APC antibody	BioLegend	Cat#328620
Rabbit monoclonal anti-human γ-tubulin antibody	Abclone	Cat#A9657
Rabbit monoclonal anti-human Phospho-PI3K antibody	Abcam	Cat#4257
Rabbit monoclonal anti-human Phospho-Gab2 antibody	Cell Signaling Technology	Cat #3882
Mouse monoclonal anti-human phospho- VAV1 antibody	R&D	Cat#MAB37861
Rabbit anti-human phospho-Akt antibody	Cell Signaling Technology	Cat#9271
Goat anti-mouse IgG H&L (Alexa Fluor® 488)	Abcam	Cat#ab150113
Goat anti-mouse IgG H&L (Alexa Fluor® 594)	Abcam	Cat#ab150116
Mouse monoclonal anti-human CD253(TRAIL) antibody	eBioscience	Cat#16-9927-82
Mouse monoclonal anti-human NKG2D antibody	eBioscience	Cat#14–5878-82
Recombinant DNA		
pLenti-Lifeact-tdTomato	Addgene	RRID: Addgene_64048
mTurquoise2-Paxillin	Addgene	RRID: Addgene_176107
ZapLck biosensor	Addgene	RRID: Addgene_131584
psPAX2	Addgene	RRID: Addgene_12260
pMD2.G	Addgene	RRID: Addgene_12259
Oligonucleotides		
KLRK1 siRNA	Invitrogen	Cat#108247
Negative control siRNA	Invitrogen	Cat#AM4641
See Table S1 for primer list		
Chemicals, peptides, and recombinant proteins		
Poly-D-lysine	Gibco	Cat#A3890401
Collagen type I	Sigma	Cat#C3867-1VL
Bovine serum albumin	VWR Life Science	Cat#0332-500G
DMEM	Gibco	Cat#11995081
MEMα	Gibco	Cat#12561056
Penicillin/streptomycin	Gibco	Cat#0378016
Trypsin-EDTA (0.25%), phenol red	Gibco	Cat#25200072
Opti-MEM	Gibco	Cat#31985062
Puromycin	Gibco	Cat#A1113803
ProLong Gold Antifade Mountant with DAPI	Invitrogen	Cat#S36938
Cell tracker deep red dye	Invitrogen	Cat#C34565
Cell tracker green CMFDA dye	Invitrogen	Cat#C2925
Recombinant human TNF alpha protein	Abcam	Cat#ab259410
Recombinant human interferon gamma protein	Abcam	Cat#ab259377
Human MICA protein	Acro Biosystems	Cat#MIA-H5253-100ug
Recombinant human ICAM-1	PeproTech	Cat#150–05
Inositol	Sigma	Cat#I7508-50G
2-Mercaptoethanol	Sigma	Cat#M3148-25ML
Folic acid	Sigma	Cat#F8758-5G
Recombinant IL-2	PeproTech	Cat#200-02
Horse serum	Sigma	Cat#H1270-100ML
Propidium iodide	Invitrogen	Cat#P1304MP
LY294002	MCE	Cat#HY-10108
Azathioprine	MCE	Cat#HY-B0256
FBS	Gibco	Cat#A3160802
Experimental models: cell lines		
NK92	ATCC	CRL-2407
MDA-MB-231	ATCC	HTB-26
Critical commercial assays		
Human interferon gamma ELISA Kit	Abcam	Cat#ab100537
Human TNF alpha ELISA Kit	Abcam	Cat#ab181421
Software		
GraphPad Prism v8.3.1	GraphPad Software	RRID: SCR_002798
ImageJ v1.41	NIH	RRID: SCR_003070
NIS-elements	Nikon	RRID:SCR_014329

### Transfection

The plasmids of Lifeact-tdTomato, mTurquoise2-Paxillin, and Lck FRET sensor purchased from Addgene were first co-transfected into HEK-293T cells with packaging and envelope plasmids. Supernatants containing viral particles were collected 36 and 72 h after transfection and filtered through a 0.45 μm filter, and the retrieved viruses were then concentrated using PEG8000 (Beyotime). Briefly, 30 ml of filtered crude lentivirus solution was added with 7.5 ml concentrating solution (50 g PEG8000 diluted in 200 ml H_2_O, added with 8.766 g NaCl). The mixture was then maintained at 4 °C overnight and centrifuged at 4000 g for 30 min to obtain the condensed viral particles. For the transfection of NK92, 2 × 10^5^ cells per well were seeded into 24-well plates and then treated with 6 *μ*M BX795 (InvivoGen) 30 min prior to the transfection. Appropriate amount of viral supernatant was added into the well along with 8 μg/ml polybrene (Beyotime). The plates were centrifuged at 1000 g for 1 h at room temperature followed by incubation at 37 °C, 5% CO_2_ for 4–6 h. Then the virus-containing supernatant was removed and replaced by fresh growth medium. For the transfection of MDA-MB-231 cells, 2 × 10^5^ cells per well were seeded into 24-well plates one day before transfection. Appropriate amount of viral supernatant was added into the well along with 8 *μ*g/ml polybrene. After 6 h, virus-containing supernatant was removed and fresh growth medium was added. Virus transfection was validated by BD FACSAria III Cell Sorter (BD Biosciences) and the transfected cells were enriched through selective drug treatment.

For the knockdown experiments, NK92 cells were transfected with siNRA (Invitrogen) targeting KLRK1 using Gene Pulser Xcell Electroporation Systems (Bio-Rad) according to the manufacturer's instructions. Briefly, 10 nM siRNA was added into NK92 cell suspension with a cell concentration of 1.5 × 10^6^/ml. Electroporation was conducted under 150 *μ*F plus 300 V for 3 pulses. After 48 h, the knockdown efficiency was validated by qRT-PCR and immunofluorescence staining.

### Shear stress treatment

The *in vitro* circulation system mainly included a peristaltic pump (P-230, Harvard Instruments, Holliston, MA, USA), a silicone microtube (diameter 0.51 mm, length 1.5 m), and a 1 ml syringe used as a reservoir for the cell solution. This system produced pulsatile flow that could simulate FSS in the blood circulation. In accordance with Poiseuille's law, the wall shear stress (dyn/cm^2^) in the tubing was calculated as τ = 4μQ/( π R^3^) where Q is the flow rate (from 0.001 to 230 ml/min), and μ is the liquid dynamic viscosity (0.01 dyn/cm^2^ for cell culture media), and R is the radius of the tube (0.255 mm). The entire system was sterilized with 75% ethanol and then rinsed with 4 ml of phosphate-buffered saline (HyClone) before the experiment. To avoid the attachment of suspended cells to the tubes and syringes, the system was pretreated with 1% bovine serum albumin (VWR Life Science). For FSS treatment, 1 ml cell suspension was added into the circulation system and subjected to different magnitudes of shear stress for 4 h at 37 °C and 5% CO_2_.

In some experiments, cells were adhered to a microfluidic chip coated with poly-D-lysin (Gibco), ICAM-1 (PeproTech), or MICA (Acro Biosystems), which was connected with the above circulation system. The cells were then treated with FSS.

### Cytotoxicity assay

To measure the cytotoxicity of NK92 against suspended MDA-MB-231 cells, these two cell types were labeled with deep red (Invitrogen) and green cell tracker (Invitrogen) before co-culturing, respectively. MDA-MB-231 cells were detached from cell culture dish with 0.25%Trypsin-EDTA (Gibco) and resuspended with full medium of MEMα. NK92 cells and MDA-MB-231 cells were then co-cultured at the ratio of 4:1 (total 1 × 10^6^ cells) for 4 h at static condition or under FSS treatment. MDA-MB-231 cells alone under static condition or under FSS were set as control groups. After co-culturing, all the cells were collected and propidium iodide (Invitrogen) was added to detect dead tumor cells. Lysis ratio (LR) of target cells was detected using BD Accuri C6 (BD Biosciences) before and after co-culturing and calculated by

LR=(1−survived target cell number/total target cell number)×100%. (1)

The increment was calculated by

$$\mathrm{Increment}=({LR}_{{NK\_\mathrm{FSS}}^{}}-{LR}_{\mathrm{Spontaneous}})-({LR}_{{NK}^{}}-{LR}_{\mathrm{Spontaneous}})-({LR}_{{\mathrm{FSS}}^{}}-{LR}_{\mathrm{Spontaneous}}).$$ (2)

The normalized increment was calculated by

$$\mathrm{Normalized}\;\mathrm{Increment}=\frac{\mathrm{Increment}}{\left({LR}_{{NK}^{}}-{LR}_{\mathrm{Spontaneous}}\right)}.$$ (3)

Target cell death was also indicated by caspase-3 activation ratio among total MDA-MB-231 cells after FSS treatment. Then, 5 μM caspase-3 substrate (Beyotime) was added to cell suspension and incubated for 20 min at 37 °C, 5% CO_2_. Then caspase-3 activation was detected by fluorescent imaging. The increment of caspase-3 positive ratio was calculated according to the formula (2).

### ELISA assay

The ELISA of TNF-α (Abcam) and IFN-γ (Abcam) was performed according to the manufacturer's instructions. Quantification was performed after collection of supernatants.

### qRT-PCR

To analyze gene expression, cells were centrifuged and then total RNAs were extracted using Aurum Total RNA Mini Kit (Bio-Rad) according to the manufacturer's instructions. RNA quality was measured and quantified using NanoDrop™ (Thermo). Complementary DNA was synthesized using Revert Aid First Strand cDNA Synthesis Kit (Thermo Fisher) according to the manufacturer's instructions. qRT-PCR was performed using RR036A PrimeScript™ RT Master Mix (TaKaRa) and CFX96 Real-Time System (Bio-Rad). The primers were obtained from the National Center for Biotechnology Information (NCBI) database and are listed in the supplementary material, Table I. For data analysis, the expressions of all genes were normalized using the ΔΔcycle threshold method against human glyceraldehyde 3-phosphate dehydrogenase (GAPDH).

### Microscope imaging and image analysis

For immunofluorescence staining, tumor cells and NK92 cells after various treatments were collected and fixed with 4% paraformaldehyde solution (PFA) (Thermo Scientific™) for 15 min. Next, 0.1% Triton X-100 (SAFC) in 1% BSA solution was used to block and permeabilize the cells for 30 min at room temperature. After washing for three times, cells were stained with Granzyme B-PE (eBioscience), CD107a-APC (BioLegend) directly, or the first antibody: γ-tubulin (Abclone), phospho-PI3K (Abcam), phospho-Gab2(Abcam), phospho-VAV1 (Abcam), phospho-Akt (Abcam) at 4 °C overnight, and then stained with the corresponding secondary antibody: goat anti-mouse IgG H&L (Alexa Fluor® 488) (Abcam) and goat anti-mouse IgG H&L (Alexa Fluor® 594) (Abcam) at room temperature for 1 h. Cells were washed with PBS for three times and immersed in ProLong Gold Antifade Mountant with DAPI (Thermo Fisher Scientific). At least 50 cells/condition were imaged by Leica TCS SPE confocal microscope. ImageJ (NIH) was utilized to analyze the fluorescence intensity.

For the FRET experiment, the images were collected with a 420DF20 excitation filter, a 450DRLP dichroic mirror, and two emission filters controlled by a filter changer (472/30 for CFP and 542/27 for FRET). The pixel-wise images of the YFP/CFP ratio were computed to quantify the FRET signals, which represent the Lck activity. A Nikon Eclipse Ti inverted microscope (Nikon H550L) with a camera (ANDOR, Zyla sCMOS) and a 60×/1.40 oil Nikon objective was used to capture and analyze all images with NIS4.1.2 software.

### Flow cytometry

The amount of granzyme B in tumor cells was analyzed using flow cytometry. MDA-MB-231 and NK92 were labeled with green and deep red cell tracker, respectively. After co-culturing, cells were fixed and permeabilized as mentioned above. Then granzyme B-PE antibody was used to stain granzyme B in MDA-MB-231 cells. After washing with PBS for three times, the granzyme B level was analyzed using BD Accuri C6 (BD Biosciences). The data were analyzed using FlowJo_v10.6.2.

### Statistical analysis

All the results were presented as mean ± SEM (standard error of the mean). The statistics between two conditions and among three or more conditions were analyzed by two-tailed Student's t-test and analysis of variance (ANOVA) if the required conditions were fulfilled (e.g., the samples assumed normal distribution), or by Mann–Whitney U-test and Kruskal–Wallis one-way ANOVA if otherwise.

## SUPPLEMENTARY MATERIAL

See the supplementary material that includes a list of PCR primers and three supplementary figures.

## Data Availability

The data that support the findings of this study are available from the corresponding author upon request.
